# Biocomposite for Prolonged Release of Water-Soluble Drugs

**DOI:** 10.3390/pharmaceutics15061722

**Published:** 2023-06-14

**Authors:** Lyghia M. A. Meirelles, Raquel de Melo Barbosa, Renato Ferreira de Almeida Júnior, Paula Renata Lima Machado, Luana Perioli, César Viseras, Fernanda Nervo Raffin

**Affiliations:** 1 Laboratory of Drug Development, Department of Pharmacy, Federal University of Rio Grande do Norte, Natal 59012-570, Brazil; lyghiamaria@unifsa.com.br (L.M.A.M.); fernanda.raffin@ufrn.br (F.N.R.); 2 Department of Pharmacy and Pharmaceutical Technology, Faculty of Pharmacy, Campus de Cartuja s/n, University of Granada, 18071 Granada, Spain; cviseras@ugr.es; 3 Immunology Laboratory, Pharmacy Faculty, Federal University of Rio Grande do Norte, Natal 59010-180, Brazilpaulamachado@ufrnet.br (P.R.L.M.); 4 Department of Pharmaceutic Science, University of Perugia, 06123 Perugia, Italy; luana.perioli@unipg.it; 5 Andalusian Institute of Earth Sciences, CSIC-University of Granada, Av. de Las Palmeras 4, 18100 Armilla, Spain

**Keywords:** chitosan, palygorskite, composite, drug delivery, prolonged release

## Abstract

This study aimed to develop a prolonged-release system based on palygorskite and chitosan, which are natural ingredients widely available, affordable, and accessible. The chosen model drug was ethambutol (ETB), a tuberculostatic drug with high aqueous solubility and hygroscopicity, which is incompatible with other drugs used in tuberculosis therapy. The composites loaded with ETB were obtained using different proportions of palygorskite and chitosan through the spray drying technique. The main physicochemical properties of the microparticles were determined using XRD, FTIR, thermal analysis, and SEM. Additionally, the release profile and biocompatibility of the microparticles were evaluated. As a result, the chitosan–palygorskite composites loaded with the model drug appeared as spherical microparticles. The drug underwent amorphization within the microparticles, with an encapsulation efficiency greater than 84%. Furthermore, the microparticles exhibited prolonged release, particularly after the addition of palygorskite. They demonstrated biocompatibility in an in vitro model, and their release profile was influenced by the proportion of inputs in the formulation. Therefore, incorporating ETB into this system offers improved stability for the administered product in the initial tuberculosis pharmacotherapy dose, minimizing its contact with other tuberculostatic agents in the treatment, as well as reducing its hygroscopicity.

## 1. Introduction

Materials science has generated a wide variety of new inputs over the recent years. Therefore, the study of interactions between biopolymers and phyllosilicates has resulted in the development of composites, which acquire properties different to those of the original organic and inorganic components. These properties consist of improvements in thermal behavior, mechanical resistance, and swelling capacity, among others [1]. The versatility of these composites is observed by the range of applications reported in the literature, among which are systems for effluent treatment, tissue engineering, biosensors, or drug delivery systems [2,3,4,5,6].

Chitosan is a cationic polysaccharide derived from chitin and is mainly extracted from the exoskeleton of crustaceans. This biopolymer is widely used in drug delivery systems due to its attractive properties, such as its biocompatibility, biodegradability, and mucoadhesiveness, in addition to being produced entirely and at a low cost for the pharmaceutical industry [7,8]. However, its limited solubility at neutral or basic pH and the high swelling and degradation in aqueous media can result in rapid drug release. Therefore, the addition of crosslinking agents in the formulation which serve to improve their stability in biological media is necessary [9].

Clay minerals have relevant properties for the preparation of composites, such as high surface area, porosity, ion exchange capacity, and silanol groups on the external surface [10,11]. Furthermore, the biocompatibility, biodegradability, low cost, and adsorptive ability of phyllosilicates make them suitable matrices for modified drug delivery [12]. Fibrous phyllosilicates, such as sepiolite and palygorskite, have a structure that favors the insertion of small molecules in their tunnels and channels, formed by the periodic inversion of the tetrahedral clay layer. In addition, the presence of large silanol groups on the surface allows the interaction of these clays with hydrophilic molecules [13,14]. In the literature, there are several studies that show phyllosilicates as drug carriers or about the preparation of biocomposites capable of modulating the release of bioactive compounds [15,16,17].

Alcantara et al. (2014) prepared composites based on palygorskite and chitosan with improved mechanical and barrier properties, thermal stability, and water resistance [18]. Previous work proposed the use of composites based on palygorskite and chitosan as drug carriers, achieving controlled release profiles, with less of a ‘burst’ effect, either by coating the inorganic fiber with the polymer or by the crosslinking impact of the clay on the polymeric matrix [19,20], offering a more significant barrier to drug diffusion from the system.

Prolonged release is interesting when one wants to simplify dosage, increase treatment adherence, and obtain less variation in drug plasma levels. However, this release profile is a challenge when modulating the dissolution of widely soluble drugs carried by hydrophilic polymers is necessary. Therefore, adding palygorskite enables slower drug dissolution [21] and provides adsorbent properties that favor the physicochemical stability of systems involving drugs and/or hygroscopic matrices [22,23].

The choice of ethambutol (ETB) is justified as follows: (1) ETB is a hygroscopic powder that acidifies the medium when it is in solution; (2) the acidic pH promotes the interaction between isoniazid and rifampicin, compromising their bioavailability; (3) due to the high aqueous solubility of ETB, it dissolves rapidly, thereby enhancing the interaction with other tuberculostatics when released immediately [24,25]. Therefore, this work aims to develop a biocomposite capable of controlled release of water-soluble drugs, using ethambutol. Hence, this study aims to overcome the high solubility of ethambutol in a solution by utilizing a polymer/clay-based system derived from natural resources for the treatment of Tuberculosis, which is still a neglected disease.

## 2. Materials and Methods

### 2.1. Materials

Palygorskite was kindly donated by Ummio Indústria de Minérios Ltd.a (extracted from São Pedro, Piauí, Brazil). This magnesium aluminum silicate was previously characterized by our group, with a specific area of 122.5 m^2^/g [26]. ETB and chitosan (with ≈91% of deacetylation degree and 185 kDa MW [27]) were obtained from Hildose (Mumbai, India) and Polymar (Fortaleza, Brazil), respectively. Dulbecco’s Modified Eagle Medium—DMEM, essential amino acids solution, and antibiotics solution (streptomycin 10 mg/mL, penicillin 10.000 UI/ mL, and amphotericin B 25 μg/ mL) were purchased from Sigma Aldrich (São Paulo, Brazil). HPLC grade methanol was purchased from JTBaker (Phillipsburg, NJ, USA). All other chemicals were of analytical grade.

### 2.2. Preparation of Biocomposites

**Figure 1:**
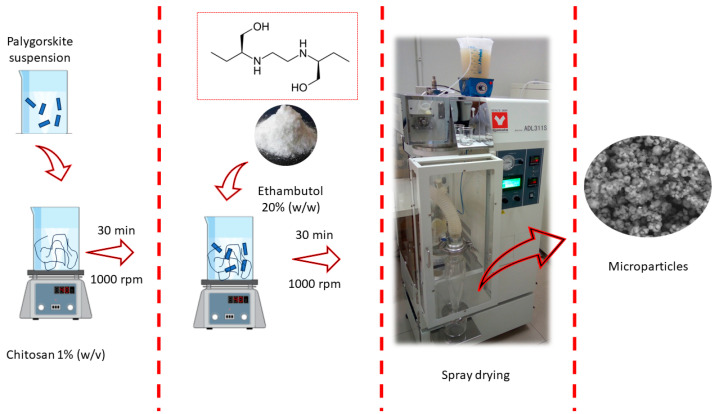
Schematic representation of the preparation of palygorskite and chitosan microparticles loaded with ethambutol.

The production of composite involved different steps and followed an adapted methodology, according to Wu and collaborators [28] (Figure 1). First, a solution of chitosan 1% (*w*/*v*) in acetic acid 1% (*v*/*v*) was prepared by constant mechanical stirring for 12 h. Next, palygorskite 1% (*w*/*v*) in acetate buffer pH 4 was dispersed in an ultra-turrax homogenizer (T18, IKA, Wilmington, NC, USA) at 1000 rpm for 30 min. Finally, 200 mL of the chitosan solution was added to different volumes of the 1% (*w*/*v*) palygorskite suspension, and the pH was adjusted to 4 with pH 4 acetate buffer to obtain a final volume of 400 mL. As a result of the final sample composition, all formulations had the same ETB mass (equivalent to 20% (*w*/*w*) of the chitosan content). The composite suspensions were dried in a spray dryer (ADL311S, Yamato Scientific Co., Tokyo, Japan) equipped with a 0.4 mm nozzle, under a flow rate of 10 mL/min, at an inlet temperature of 150 °C, outlet temperature between 95–100 °C, and an atomization pressure of 0.1 atm. Table 1 represents the code adopted for each formulation, its respective composition, and yield.

### 2.3. Characterization of Biocomposites

Laser granulometry determined particle size using Cilas 1090 (Orleans, France). The measurements were carried out in dry mode, depositing each sample in the appropriate device under a frequency of 55 Hz and pressure of 2500 Mb. The results represent the mean diameter (μm) of the particles. For ETB quantification, approximately 10 mg of the particles was dispersed in 5 mL of a 0.25% (*v*/*v*) acetic acid solution, pH 3.2. The samples were agitated for one hour and then centrifuged at 4000 rpm for 20 min. The supernatant was collected and filtered using a 0.45 μm membrane for drug quantification. ETB concentrations were determined by HPLC coupled with a Variable Wavelength Detector (1260 Infinity II, Agilent, Santa Clara, CA, USA) according to the methodology of Zhao et al. [29]. The mobile phase was acetate buffer at pH 5.0 and methanol (83:17). The flow rate and the injection volume were 1 mL/min and 10 μL, respectively. The UV absorbance was measured at a wavelength of 270 nm and at a resolution of 2 cm^−1^. The stationary phase was a Generix 5 C18 column (4.6 mm × 150 mm, 5 μm) (ACE, Scotland, UK). Encapsulation efficiency was calculated by the percentage of ETB determined in the microparticles according to the amount of drug added to the preparation (Equation (1)). Drug loading was determined by the rate of ETB concerning microparticle mass (Equation (2)).

(1) $$Encapsulation\;efficiency\;(\%)=\frac{Mass\;of\;drug\;in\;microparticles}{Mass\;of\;drug\;added\;to\;formulation}\times 100$$

(2) $$Drug\;loading\left(\%\right)=\frac{Mass\;of\;drug\;in\;microparticles}{Mass\;of\;microparticles}\times 100$$

Zeta potential (ζ) values were determined at 25 °C using Zetasizer (Nano ZS, Malvern Instruments, Worcester, UK). The samples were suspended in distilled water at a concentration of 1% (*w*/*v*), and each measurement obtained corresponds to the average of 15 runs, which were performed in triplicate for each sample. With Scanning Electron Microscopy (SEM), it was possible to evaluate the morphology of the composites obtained. The composites were deposited on a carbon tape and analyzed at 5 kV under a microscope (TM3030, Hitachi, Tokyo, Japan). A diffractometer (XDR-6000, Shimadzu, Kyoto, Japan) with Cu kα radiation at a voltage of 40 kV and current of 30 mA, 5–40° (2θ) determined the crystallographic screening of the samples. FTIR spectra were obtained using a spectrometer (IRPrestige-21, Shimadzu, Kyoto, Japan) by attenuated reflectance, with 32 scans and a resolution of 4 cm^−1^, in an interval of 700–4000 cm^−1^. Thermogravimetric analysis (TG) and Differential Scanning Calorimetry (DSC) were performed in a thermogravimetric analyzer (TGA/DSC 3+, Mettler Toledo Co., Greifensee, Switzerland) equipped with thermosensors and a microbalance. The samples were heated in an atmosphere of synthetic air, with a flow of 100 mL/min, under a heating rate of 10 °C/min, in the range of 30–950 °C (TG) and 30–400 °C (DSC).

### 2.4. In Vitro Release Kinetics

The ETB release profile from the particles was determined using the 6–8 kDa cut-off dialysis membrane method (Spectra/Por^®^1, Spectrum Labs, San Francisco, CA, USA). The assay was carried out in two different media, phosphate buffer pH 2.2 and phosphate buffer pH 6.8, maintained at 37.0 ± 1.0 °C, under 150 rpm, in an incubator with orbital agitation (Q315IA, Quimis, Diadema, Brazil). First, a quantity of composites corresponding to 10 mg of ETB was added to the dialysis tube and suspended in the appropriate medium. The tubes were then immersed in 30 mL of the medium, ensuring the sink condition would be reached. Next, aliquots of 1 mL of the medium were collected at determined intervals, replacing an equal volume of the fresh medium after each collection. The ETB concentration in each aliquot was determined according to the previously described methodology. In addition, each formulation was analyzed in triplicate, and data were plotted using the percentage of drug released cumulatively as a function of time.

To determine the mechanisms involved in drug release from the biocomposites, model-dependent methods were tested using the Microsoft Excel add-in DDsolver. The selection of the presented models was based on the best adjustments of the obtained data, considering the correlation coefficient. Release profiles of drug from microparticles were analyzed by applying Higuchi’s kinetics (cumulative percent drug release vs. square root of time), the Korsmeyer–Peppas equation (log cumulative percentage of drug release vs. log time), and Peppas–Sahlin (relaxation/Fickian ratio vs. fraction released). The equations and parameters evaluated in each model are described in Table 2.

*Q_t_* corresponds to the amount of drug released in time *t*; *K_H_* is the Higuchi dissolution constant; Mt/M∞ corresponds to the fraction of drug released as a function of time *t*; *K_P_* is a constant related to the geometry and structure of the system; *n* is the release exponent (indicates the release mechanism); *K*_1_ and *K*_2_ represent the constants related to the contributions of the Fickian diffusion mechanism and the erosion/relaxation mechanism (Case II transport), respectively. The coefficient ‘*m*’ is the Fickian diffusion exponent of the modified-release system, whatever its form. The type of transport involved in the system can be determined from the ‘*n*’ parameter and generally comprises Fickian (the drug concentration gradient directs release) and non-Fickian (transport is governed by swelling or relaxation polymeric chain) [32].

### 2.5. In Vitro Cytotoxicity Assay

Cytotoxicity evaluation was based on a quantitative colorimetric determination using the 3-(4,5-dimethylthiazol-2-yl)-2,5-diphenyltetrazolium bromide salt, known as MTT [33]. Metabolically active cells have mitochondrial dehydrogenases capable of converting MTT into formazan, which generates a color change proportional to the number of viable cells [34]. BALB/3T3 clone A31 embryonic fibroblasts from mice were purchased from the Rio de Janeiro Cell Bank (BCRJ). Cells from the 3T3 cell line were cultured in Dulbecco’s Modified Eagle’s Medium (DMEM) at 37 °C in an air atmosphere (5% CO_2_). Plating was performed in 96-well microplates, followed by incubation in a humid air atmosphere (5% CO_2_) at 37 °C and for 24 h. After the culture period, the culture medium was removed and replaced by 200 μL of some dilutions (12.5–100 μg/mL, concerning ETB) of the samples in the appropriate culture medium. Then, the microplate was subjected to another incubation period of 72 h. After this, the cells were treated with 50 μL of the MTT solution and incubated for an additional 4 h. Finally, 100 μL of DMSO was added to each well to ensure the complete dissolution of the formazan crystals that were produced after the enzymatic reduction of MTT. The microplates were then subjected to agitation to homogenize the content, and then the absorbances were determined in a microplate reader (Epoch, Biotek, Winooski, VT, USA) at a wavelength of 540 nm.

### 2.6. Statistical Analysis

A two-way ANOVA model statistically analyzed the cell viability of the obtained systems, followed by the Tukey post-test with multiple comparisons, to determine the difference between the analyzed samples at a significance level of *p* < 0.05, using the Prism 6.01 software (GraphPad Software 2012, San Diego, CA, USA).

## 3. Results and Discussion

### 3.1. Characterization of Biocomposites

All samples showed yields of between 50 and 60%, which corroborates with other published works that used a similar methodology for producing chitosan microparticles on a small scale [35,36,37,38]. The loss during drying must be related to the exhaustion of a fraction of the particles with significantly reduced size, a direct consequence of the low concentration of chitosan used in this work, in addition to the adhesion of the material to the walls of the drying chamber of the equipment. Phyllosilicates are materials recognized for their desiccating properties, being stable against relative humidity, and providing good flow characteristics to the materials that they make up [39]. These characteristics justify the slightly higher performance of the composites compared to chitosan microparticles.

Table 3 shows the results related to the properties of the particles obtained. The mean particle diameter remained within the micrometer range (3–5 µm). The increase in proportion of palygorskite in the material composition caused a slight increase in the average diameter of the product obtained, since the total concentration of dispersed solids increased from CE to C1P1E. The presence of palygorskite caused flocculation of the chitosan chains, entangling the phyllosilicate’s fibrous structure, as Rusmin et al. (2015) indicated [40].

Chitosan/palygorskite microparticles loaded with sodium diclofenac and prepared by the same technique showed an almost similar mean diameter, although a larger diameter nozzle and a higher polymer concentration were used in this example [20]. It is possible that the addition of the covalent crosslinker, glutaraldehyde, caused the formation of a denser agglomerate of the polymeric matrix, despite the differences mentioned above [41].

C2P1E and C1P1E did not have any crosslinker added other than palygorskite, which offers less risk of toxicity against chemical agents, such as glutaraldehyde [42]. The ability of phyllosilicates to form points of interaction between hydrophilic polymeric chains was demonstrated in previous works [40,43].

The binding capacity shown in Table 3 was slightly higher than the values obtained by Khlibsuwan et al. (2017) when preparing hybrid microparticles of chitosan and montmorillonite loaded with propranolol hydrochloride using the same technique [28]. The addition of palygorskite had a detrimental effect on the amount of ethambutol in the composites. However, the moderately negative surface charge of the inorganic carrier could retain the drug, as was demonstrated in previous works [15]. Since chitosan and drug concentration maintain proportionality, ethambutol and phyllosilicate compete for the same hydrogen bonding sites as the polymer.

The interaction of palygorskite with chitosan is possible through hydrogen and electrostatic bonding between the chemical species, involving the surface of the phyllosilicate with the polymeric chain [44]. In this way, palygorskite neutralizes the polymer’s positively charged amino groups, minimizing the macromolecular chain’s electrostatic repulsion, making it less distended. This alteration implies a smaller interaction of chitosan with the drug dissolved in the medium [45].

The microparticles obtained showed positive zeta potential values, given the cationic character of the polymer that makes up the matrix of the system and the drug carried (Table 3). In addition, the electropositive surface of composites based on chitosan and palygorskite confirms the coverage of inorganic particles by the polymer’s ionized chain.

The C2P1E and C1P1E composites, in turn, showed a reduction in the surface charge concerning the chitosan microparticles, due to the electrostatic interaction between the negatively charged clay mineral and the cationic polyelectrolyte, with partial neutralization of the chitosan amino groups. However, the increase in the palygorskite concentration did not cause a significant change in the surface charge of the composites, because at the pH of the water used as a dispersant medium, there is a more considerable ionization of the chitosan chains (pKa 6.5). At the same time, the clay mineral had its zeta potential reduced by the presence of excess H^+^ adsorbed on its surface, thus exerting less influence on the charge of the composite [46].

**Figure 2:**
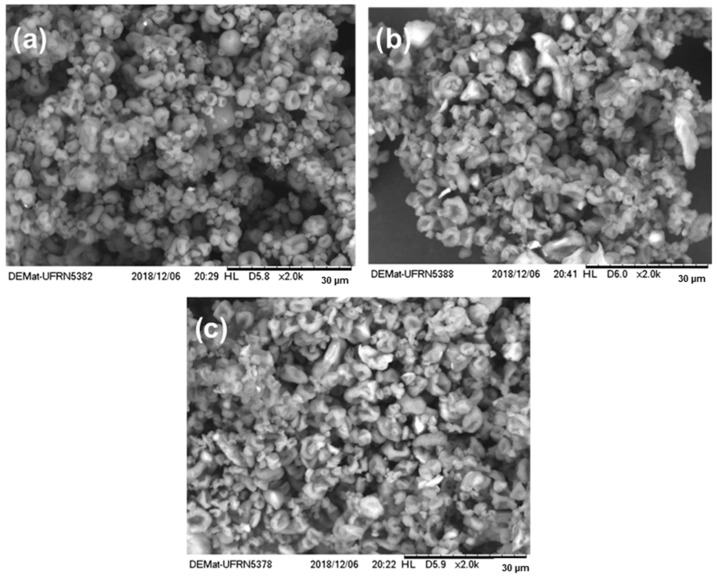
Electron micrograph of chitosan–ethambutol CE (**a**), chitosan–palygorskite–ethambutol C2P1E (**b**), and C1P1E (**c**) microparticles.

Figure 2 demonstrates the morphology of the microparticles by the electromyography technique. It is possible to observe that, under the conditions employed, the particles presented a rounded shape with a concave surface. The depression on one side of the microparticles may be related to the reduced viscosity of chitosan at the concentration used [41]. Therefore, when exposing the droplet formed by the spray nozzle to a high temperature, the rapid evaporation of the solvent caused deformation in the particle due to the inability of the polymer to maintain the initially formed structure.

As the proportion of palygorskite in the formulation increased, a tendency to deform microparticles was observed. This can be explained by the fibrous morphology of palygorskite and chitosan’s capacity to give plasticity to the system, which allows more spherical particles to form.

**Figure 3:**
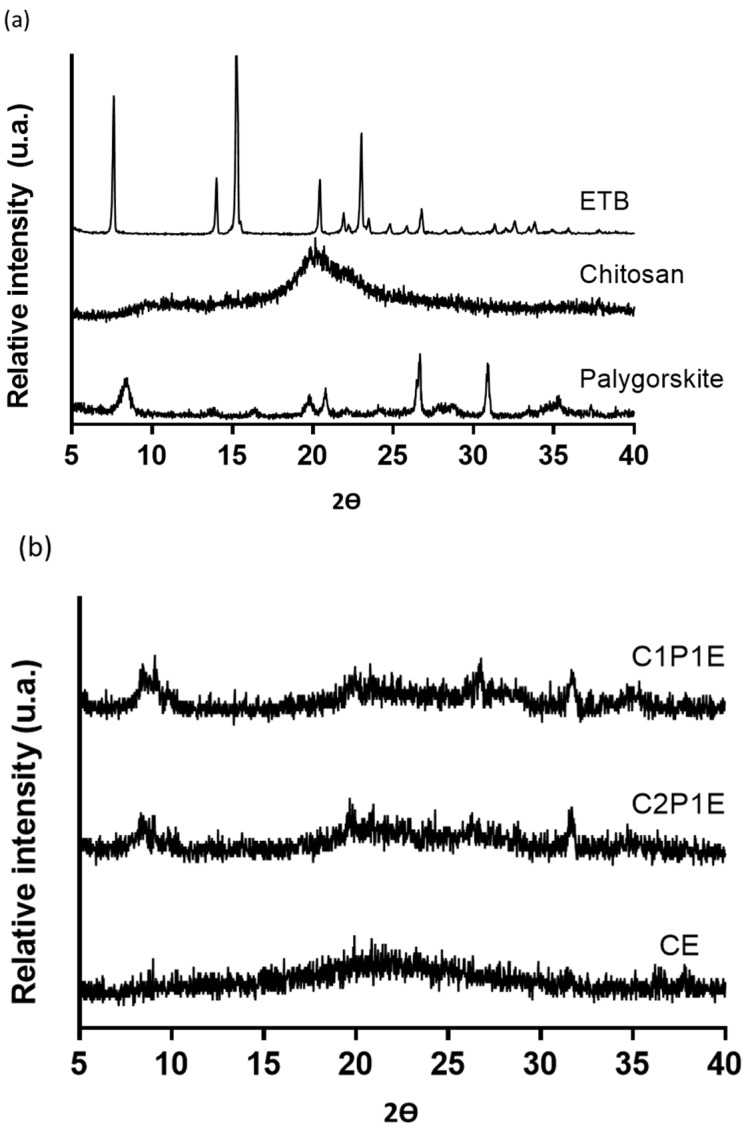
X-ray diffractograms of raw materials (**a**) and microparticles (**b**).

Figure 3a shows diffractograms of the starting materials, the crystallinity of the drug (2θ = 7.6°, 14.0°, 15.2°, 20.4°, and 21.9°) and of the clay mineral (2θ = 8.3°, 19.7°, and 20.8°) [47,48]. On the other hand, chitosan is a semicrystalline polysaccharide that showed a broad peak around 2θ = 20°. This characteristic relates to the degree of deacetylation and the treatment to which the material underwent [49]. After spray drying, the crystallographic profile of the microparticles indicated their amorphous character (Figure 3b). The mechanism that triggers this transition is based on the external energy supply through the rapid evaporation of the solvent in which the drug was dissolved [50]. Many works evaluated the preparation of micro- and nanoparticles from chitosan polymer [51,52,53]. These particles showed loss or reduction of the 2θ = 20° peak, with suppression of the crystalline reflections of the added drugs, in most cases. The observed amorphous state suggests homogeneous drug distribution in the system, which influences its release profile and is reflected through the new interactions demonstrated in the FTIR spectrum.

The ethambutol peak was not identified in the diffractograms for all samples (CE, C2P1E, and C1P1E) due to its conversion into an amorphous form when interacting with other compounds of microparticles. The same behavior was observed by Wu et al. (2014) with chitosan/palygorskite microspheres loaded with sodium diclofenac by emulsification [54].

**Figure 4:**
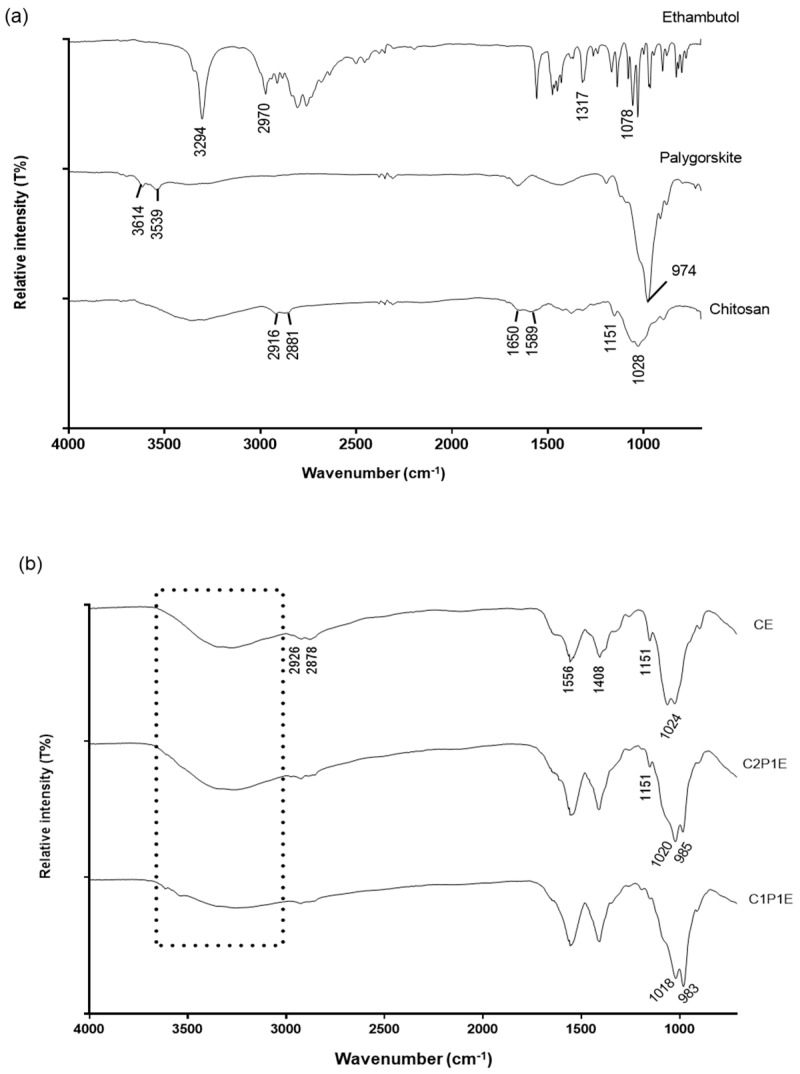
FTIR spectra of raw materials (**a**) and ETB-loaded microparticle formulations (**b**).

The interaction between components was evaluated through spectroscopic analysis of the prepared composites (Figure 4). The drug-related bands (Figure 4a) are characterized by O-H (3294 cm^−1^), N-H (2970 cm^−1^), C-N (1317 cm^−1^), and C-C (1078 cm^−1^) stretching vibrations [55]. Due to the reduced proportion of ETB in the particles, these bands are overlapped by the chitosan and palygorskite bands in the analyzed composites (Figure 4b).

In addition, chitosan showed a broad band between 3000 and 3500 cm^−1^, which refers to the polysaccharide’s N-H and O-H stretching vibrations. The amine and amide groups of chitosan are represented by the strain vibration bands at 1589 and 1650 cm^−1^, respectively [19]. Palygorskite showed bands at 3614 cm^−1^ (Al-Al-OH) and 3539 cm^−1^ (Fe-Mg-OH, Al-Mg-OH, or Fe_2_-OH), related to stretching coordinated water hydroxyls; Si-OH stretching vibration was observed at 974 cm^−1^. This palygorskite spectroscopic profile agrees with other authors’ reports [56,57].

In the spectra represented in Figure 4b, there was an overlap of the amine and hydroxyl bands of chitosan to the coordinated water bands of palygorskite in the regions of 3000–3600 cm^−1^, with a broadening and loss of band intensity as the proportion of palygorskite in the composite increased, probably ascribed to hydrogen bonding between chitosan hydroxyls and amino and palygorskite silanols, as described by Rusmin et al. (2015) [40]. At 2878 and 2926 cm^−1^, stretching bands of the -CH_3_ and -CH_2_ aliphatic groups were observed, respectively, confirming the presence of organic material in the samples. After chitosan dissolution in acetic acid and the subsequent drying process, the formation of chitosan acetate salt was observed through the emergence of two new bands, one close to 1400 cm^−1^ and the other at 1556 cm^−1^, related to the symmetric and asymmetric stretching of the carboxyl anion, to the detriment of the N-H strain bands at 1589 and 1650 cm^−1^ [58].

**Figure 5:**
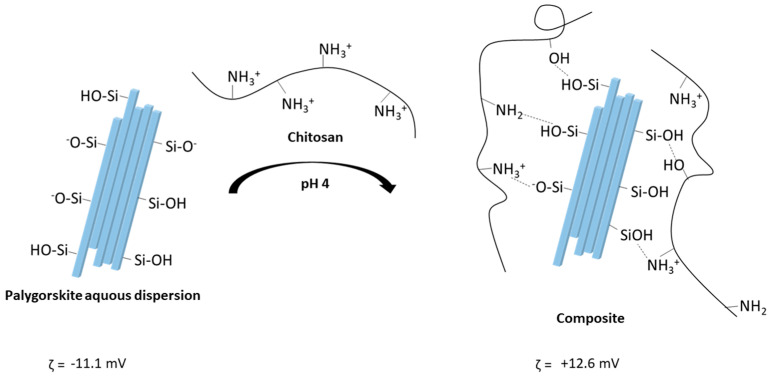
Schematic representation of palygorskite and chitosan interactions at pH 4.0.

Figure 4b shows the spectra of composite with subtle changes in the 900–1100 cm^−1^ region due to the addition of palygorskite. The band refers to primary alcohol, observed in chitosan microparticles at 1024 cm^−1^, and was slightly shifted to 1020 and 1018 cm^−1^ in C2P1E and C1P1E composites, respectively, corroborating the involvement of this group in the interaction between the components of the microparticles. The Si-OH stretching vibration, attributed to the palygorskite tetrahedral layer at 974 cm^−1^, shifted to higher wavelengths in the composites, indicating that the silanol groups are also implicated in the interaction with the organic phase of the composite, intensifying in the most abundant composite in palygorskite (C1P1E). Figure 5 shows schematic representation of interactions of palygorskite with chitosan.

**Figure 6:**
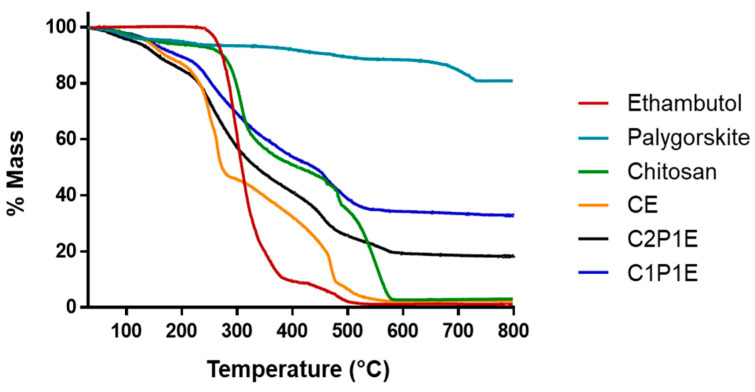
Thermogravimetric profile of microparticles and raw materials.

Figure 6 shows the thermogravimetric analysis of the microparticles obtained and the raw materials used. Below 200 °C, the adsorbed water is lost, except for samples containing only ethambutol. The chitosan TG curve shows, in addition to water loss up to 200 °C, degradation and deacetylation (200–450 °C) and oxidation of carbonaceous residues, such as dolomite (450–700 °C), corroborating the data in the literature [59].

The formation of chitosan acetate in the microparticles can alter the system’s thermal profile, causing two stages of mass loss up to 200 °C, as demonstrated by Nunthanid et al. (2004) [58]. In addition, the decomposition of the chitosan salt is more intense than that of chitosan at temperatures below 250 °C as the acetate residue induces matrix degradation [60]. The thermogravimetric profile confirmed these findings regarding microparticles, especially the CE sample, which was less thermally stable than the polymer between 100 and 250 °C.

TG curves related to the C2P1E and C1P1E composites demonstrated the displacement of the decomposition stages to higher temperatures, in addition to the attenuation of the decomposition ramp. These changes are attributed to the presence of palygorskite, providing greater thermal stability as the mass ratio of palygorskite in the sample increases. Other works show an improvement in the thermal properties of composites based on chitosan and clay minerals [61,62,63]. The events concerning polymer and ETB decomposition are completed at approximately 600 °C. The absence of any EC-related residue confirms its exclusively organic composition when compared to the C2P1E and C1P1E composites, whose mass losses of 81.7% and 67.3%, respectively, are close to the proportions of organic matter used in obtaining the particles.

**Figure 7:**
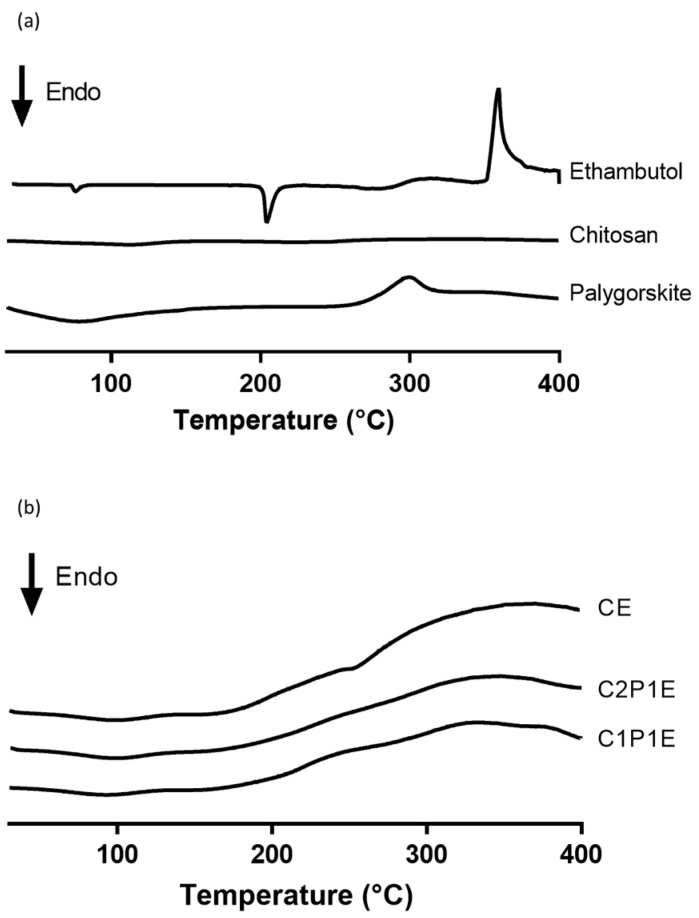
DSC curves of raw materials (**a**) and microparticles (**b**).

The DSC curve of ETB showed an endothermic peak at 205 °C, referring to melting point, which is characteristic of S,S-diastereoisomer and has a tuberculostatic effect. However, polymorphic conversion (form II to form I) is observed in a discrete endothermic peak at 76 °C (Figure 7a), as already described by Rubin-Preminger et al. (2004) [64]. In the same figure, the exothermic peak at 358 °C relates to the decomposition of ethambutol, as seen in the TG curve. The chitosan DSC curve shows an endothermic peak around 102 °C and an exothermic peak close to 295 °C. According to Lopez et al. (2008), the first is associated with adsorbed water loss, and the second is associated with polymer decomposition [65]. Palygorskite showed no significant events, which are attributed to dehydration of the adsorbed and coordinated water in the evaluated temperature range. Figure 7b demonstrates a similar thermal profile between microparticles. A discrete endothermic peak below 100 °C is related to the evaporation of adsorbed water, typical for all particle curves. The irrelevant intensity of the signal indicates that the samples were adequately dried during preparation, with no significant moisture residue remaining.

Figure 7b indicates that ethambutol is dispersed in the nanoparticle matrix, confirming the data presented in the diffractometric analyses. In addition, thermal decomposition of chitosan acetate is accompanied by an exothermic event at approximately 300 °C through the contraction of already dehydrated polymeric chains [52]. All microparticles showed this event. However, the intensity is related to the proportion of polymer in the system.

### 3.2. In Vitro Release Kinetics

**Figure 8:**
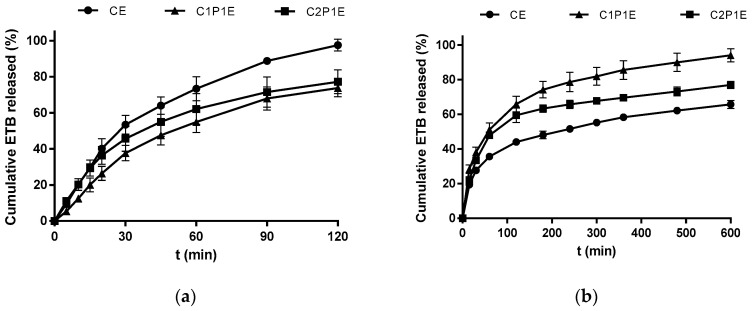
ETB release profile from microparticles at pH 2.2 (**a**) and pH 6.8, (**b**) (*n* = 3; mean ± SD).

The ETB release profiles from the microparticles are shown in Figure 8. The model drug dissolves quickly in the media tested in its free form, as it belongs to class III of the Biopharmaceutical System Classification (high solubility and low permeability) [15,66]. Thus, the developed system could be applied to other drugs with the same classification and require a reduced initial release, as observed in microparticles loaded with ETB.

The polymer: phyllosilicate ratio influenced the release profile of the hydrophilic drug from the matrix as a function of the pH of the medium since chitosan is a cationic polymer that dissolves in a dilute acid medium due to the protonation of the amine groups of the polymer, which leads to destabilization of the polymeric network of microparticles [67].

The behavior shown by chitosan microparticles (CE) confirmed the expectation that, at pH 2.2, this system would cause an intense initial release of ETB (~50%) in 30 min, with a complete release of the drug after 2 h. This change concerning the free drug may be associated with the formation of a polymer gel layer at the interface with the dissolution medium, which slows down the ETB diffusion due to the saturation of this viscous layer, as demonstrated by Alhalaweh and collaborators when analyzing microparticles of chitosan-based zolmitriptan [68].

The drug release rate reduced as the palygorskite ratio increased in an acid medium (pH 2.2), with about 50% of the drug released after the first hour. The reduction in the intensity of the initial release is more notable in the composite whose proportion of clay and polymer is equivalent, resulting in a reduction of approximately 25% in ETB released, compared to chitosan microparticles. In addition, matrices with up to 30% palygorskite showed reduced swelling, as this property is negatively correlated with the degree of crosslinking of the polymer [43]. The reduced mobility of the polymeric chains and the lower water adsorption caused by crosslinking explain the behavior of the C1P1E and C2P1E composites. However, the release at pH 6.8 was less intense in the CE sample, whose matrix was exclusively composed of chitosan. This result is attributed to the reduced solubility and protonation of the chains, given that the pKa of the polymer is slightly lower than the pH of the medium in which the test was carried out. Under this condition, the composites tend to release ETB more quickly as the concentration of chitosan in the matrix is reduced (C1P1E > C2P1E > CE).

Previously, it was demonstrated that the active release from the phyllosilicate is not significantly altered by the pH change (1.2 and 6.8), with rapid diffusion of ETB in both. Therefore, the polymer presents itself as a limiting element for the release of approximately 66% (CE), 77% (C2P1E), and 94% (C1P1E) of the drug after 10 h of testing.

The obtained data were adjusted to some mathematical models to determine the mechanisms involved in releasing the model drug from the microparticles. Drug release from hydrophilic polymer matrices can be controlled by various factors, such as polymer swelling, diffusion of the drug through the swollen polymer layer, and/or erosion of the swollen polymer [32].

In this study, it was identified that the Higuchi, Korsmeyer–Peppas, and Peppas–Sahlin mathematical models presented the best correlation coefficients; therefore, they were selected, while the zero order and first order models were excluded due to the r^2^ values < 0.6 obtained in the fit. In order to comply with the prerogatives of the Korsmeyer–Peppas model, the data used in the adjustment to this equation corresponded to the portions of the curves where the cumulative quantity dissolved was <60%. Table 4 shows the parameters obtained for the release test performed at pH 2.2.

Under this experimental condition, the Higuchi model fit better to the release of ETB from CE and C2P1E microparticles (r^2^ > 0.96), while the release of ETB from C1P1E fit better to Peppas–Sahlin (r^2^ = 0.960). Adding palygorskite reduces the release rate (*K_H_*) through the Higuchi model, possibly due to the cross-linking nature of the clay mineral, which makes the diffusion of the drug more complex through the swollen matrix in an acid medium.

Composites based on chitosan and different phyllosilicates are suitable for obtaining new platforms for drug release, due to changes in the surface charge of the hybrid, to the increase in mechanical resistance provided by the addition of the inorganic phase to the matrix, or even to a reduction in the swelling capacity of the polymer [28,54,69,70].

The values of *n* (0.45 < *n* < 0.89) obtained using the Korsmeyer–Peppas model indicate anomalous transport kinetics in all systems, that is, a combination of the two mechanisms: diffusion and Case II transport [71]. The Peppas–Sahlin parameters corroborate the anomalous transport of the asset, predominantly guided by diffusion (*K*_1_ > *K*_2_). The parameter ‘m’ also confirms this profile, given that all systems presented 0.43 < *m* < 0.85, indicative of anomalous transport in spheres [72,73].

In the data set presented in Table 5, it was observed that the adjustment was altered due to the pH change of the dissolution medium. This diverse behavior is attributed to predominantly electrostatic interactions between the materials that make up the system, a cationic polymer, a phyllosilicate with a moderately negative charge, and an ionizable drug.

Chitosan and palygorskite hybrid microspheres prepared to carry sodium diclofenac adsorbed to their surface showed similar behavior in terms of their release kinetics. Although experimental data indicate a better fit to the Korsmeyer–Peppas equation, it is also suggested that Fickian diffusion was the driving mechanism of drug release from the system at pH 6.8 [20]. Likewise, composites based on these inputs intended to release aminosalicylic acid indicated that the diffusion mechanism was predominant in the system, which better fits the Korsmeyer–Peppas model [74].

Therefore, incorporating ETB in the systems that regulate its release, especially in the initial portion of the gastrointestinal tract, where the pH favors interactions with other tuberculostatic drugs, is a relevant strategy for improving the bioavailability of the drugs. Added to this are the obvious gains that result from reduced frequency of administration, which in turn impacts on greater adherence by those patients who are subject to tuberculosis therapy.

The best fit was achieved using the Peppas–Sahlin equation in all systems, with r^2^ values > 0.98. At pH 6.8, diffusion was observed as the primary release mechanism of ETB from microparticles. The KP values are increasing in the following order: CE < C1P1E < C2P1E, indicating that reducing the polymer proportion in the formulation leads to a higher release rate. Values of *n* > 0.45 determine that the release mechanism is anomalous, corroborated by the Peppas–Sahlin model. In this medium, the polymer showed irrelevant swelling and/or erosion, given the negative value of the constant K_2_. This alteration in the release kinetics is because the chitosan chains undergo protonation at a much lower intensity at pH > pKa; therefore, they neither repel each other nor swell or dissolve.

### 3.3. In Vitro Toxicity Assay

**Figure 9:**
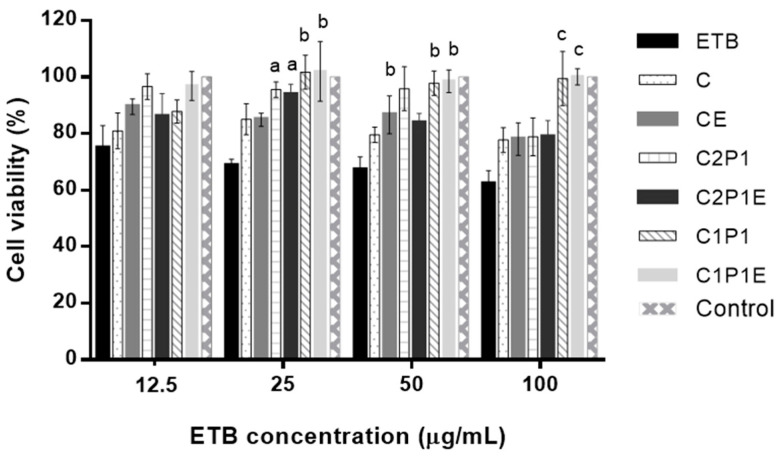
Cytotoxicity of the 3T3 strain after 72 h to different concentrations of ETB. Data are represented as mean ± standard deviation (*n* = 5). *p* < 0.05 (a); *p* < 0.01 (b); *p* < 0.001 (c), in relation to the drug. Legend: ETB—ethambutol; C—chitosan microparticle; CE—ETB-loaded chitosan microparticle; C2P1—chitosan–palygorskite (2:1) microparticle; C2P1E—ETB-loaded chitosan–palygorskite (2:1) microparticle; C1P1—chitosan–palygorskite (1:1) microparticle; C1P1E—ETB-loaded chitosan–palygorskite (1:1) microparticle.

An essential step for developing new drug delivery systems corresponds to safety when administered in the biological environment. For this purpose, cytotoxicity assays evaluated the biocompatibility of microparticles in two cell lines. The choice of concentrations used in this assay was based on the previously published minimum inhibitory concentration of ETB against strains of mycobacteria [75]. The authors reported that concentrations greater than 5 µg/mL of this anti-TB resulted in the total inhibition of bacterial growth. Therefore, in order to demonstrate the safety of the developed systems, the concentrations used in the test were 12.5–100 µg/mL. The results obtained in the cytotoxicity assay of microparticles in the 3T3 cell line are shown in Figure 9.

Drug toxicity showed no statistical difference between the evaluated concentrations, despite a tendency to reduce the number of viable cells with increasing anti-TB concentration. The cell viability of ETB (3T3) was approximately 70%, a value close to that detected by other authors under similar experimental conditions, corresponding to 85% viability [76].

Chitosan is a polymer recognized for its properties that are favorable to biological applications, such as biocompatibility, absence of toxicity, biodegradability, and mucoadhesiveness [77,78,79]. All microparticles prepared with chitosan (C and CE) showed higher viability than the free drug, indicating good biocompatibility of the carrier used.

The cytotoxic effect of polycations, such as chitosan, is mainly related to the interaction of polycations with the cell membrane, involving electrostatic interactions between the cationic macromolecules and the negatively charged membrane. These interactions correlate with chain length and the arrangement of cationic charges [80]. It is worth noting that cytotoxicity assays performed on Caco-2 and 16HBE14o-Cells strains confirmed that the polymer charge density is related to the toxic effects of chitosan, which are more intensely observed at the pH where the chain is in the ionized form [81].

The analysis of microparticles without ETB and loaded with the drug did not indicate any significance between the viability percentages, considering the increasing dilutions within the same group. In a previous study carried out by Salcedo et al. (2012), biocomposites based on chitosan and montmorillonite also did not differ significantly under the different concentrations evaluated in Caco-2 cells, even using concentrations in a larger range (5–500 µg/mL) [2].

Chitosan and palygorskite are biocompatible raw materials. Furthermore, several studies have shown the safety of using these phyllosilicates in in vitro assays using different cell lineages, such as macrophages, fibroblasts, renal epithelium, and human cervical cancer cells [82,83,84]. In addition, palygorskite has its intended use as an adjunct in treating diarrhea and as a pharmaceutical excipient in non-parenteral formulations in American and European pharmacopeias [85,86].

In samples at similar concentrations, a significant difference was identified between some of the composites and the drug, starting from 25 µg/mL, with an increase in the significance level at higher concentrations. In the literature, there are reports of superior biocompatibility of composites based on chitosan and clay minerals when compared to chitosan alone in in vitro models using 3T3 cells [87]. This can be related to palygorskite reducing the polymeric load, minimizing its interaction with the cell membrane.

The most significant differences occurred in composites C1P1 and C1P1E. Thus, phyllosilicate positively influences cell proliferation. Dumas e Pagé (1986) demonstrated the low toxicity of palygorskite samples, even at high concentrations, against 3T3 cells. According to the authors, 200, 500, and 1000 µg/mL caused 11, 26, and 28% cytotoxicity, respectively [82].

A study with Vero cells evaluated the effects of palygorskite on cell morphology and viability [88]. The images obtained demonstrated a proliferative effect of the palygorskite on the cells, corroborated by data obtained in the MTT assay. In addition, it is possible to observe that all systems with and without the drug showed cell viability more significant than 75% at the tested concentrations (Figure 9), with a considerable increase in biocompatibility in the composites with the increasing proportion of palygorskite, although in vivo tests are necessary to confirm these preliminary results.

## 4. Conclusions

The variation in the proportion of palygorskite influenced parameters such as binding efficiency, surface charge, and morphology of microparticles, which showed good dispersion of the drug in the matrix, with amorphization of the same, according to crystallographic data and DSC curves. The microparticles showed pH-dependent release and adding phyllosilicate reduced the initial release of ETB observed in chitosan microparticles. These data suggest a greater crosslinking of the polymer, limiting the diffusion of the hydrophilic drug. Depending on the proportion of chitosan and palygorskite in the matrix, the release kinetics from the microparticles fit well with the Higuchi and Peppas–Sahlin models. The composites evaluated in in vitro models were biocompatible in the 3T3 cell line, with a positive effect of the phyllosilicate on the cell viability of the microparticles. This data set allows us to envision the application of these carriers based on palygorskite and chitosan as a simple and low-cost alternative for overcoming the technical difficulties related to the need to release water-soluble drugs in a controlled manner.

## Figures and Tables

**Table 1:** Identification of microparticles, composition and yield obtained.

Formulation	Chitosan: Palygorskite (*w*/*w*)	Yield (%)
CE	1:0	52.05
C2P1E	2:1	59.37
C1P1E	1:1	58.98

**Table 2:** Kinetic models applied to evaluate the data obtained [30,31].

Models	Equation	Parameters
Higuchi	Qt=KH.t12	*K_H_*, r^2^
Korsmeyer–Peppas	$\frac{Mt}{M\infty }=K_{P}t^{n}$	*K_P_*, *n*, r^2^
Peppas–Sahlin	$\frac{Mt}{M\infty }=K_{1}t^{m}+K_{2}t^{2m}$	*K*_1_, *K*_2_, *m*, r^2^

**Table 3:** Physicochemical properties of composites based on palygorskite and chitosan (mean ± SD, *n* = 3).

Formulation	Diameter (µM)	Drug Loading (%)	Encapsulation Efficiency (%)	Zeta Potential (mV)
CE	3.17 ± 0.46	12.34 ± 0.04	94.59 ± 0.34	34.93 ± 0.99
C2P1E	3.36 ± 0.22	11.69 ± 0.18	99.39 ± 1.56	26.53 ± 3.65
C1P1E	4.29 ± 0.16	7.66 ± 0.07	84.30 ± 0.79	23.27 ± 2.87

**Table 4:** Kinetic parameters obtained from ETB release curves from microparticles at pH 2.2.

Model	Parameters	CE	C2P1E	C1P1E
Higuchi	*K_H_* *r* ^2^	9.061 ± 0.262 0.966 ± 0.015	7.568 ± 0.857 0.974 ± 0.018	6.709 ± 0.613 0.956 ± 0.002
Korsmeyer–Peppas	*K_P_* *n* *r* ^2^	4.035 ± 0.766 0.699 ± 0.079 0.860 ± 0.120	6.829 ± 1.508 0.521 ± 0.060 0.948 ± 0.036	2.245 ± 0.030 0.735 ± 0.009 0.896 ± 0.056
Peppas–Sahlin	*K*_1_ *K*_2_ *m* *r* ^2^	8.320 ± 0.636 0.422 ± 0.048 0.450 ± 0.000 0.958 ± 0.022	8.842 ± 1.652 0.068 ± 0.158 0.450 ± 0.000 0.968 ± 0.021	5.040 ± 0.891 0.481 ± 0.023 0.450 ± 0.000 0.960 ± 0.012

**Table 5:** Kinetic parameters obtained from ETB release curves from microparticles at pH 6.8.

Model	Parameters	CE	C2P1E	C1P1E
Higuchi	*K_H_* *r^2^*	3.207 ± 0.087 0.848 ± 0.024	3.936 ± 0.029 0.767 ± 0.072	4.711 ± 0.274 0.843 ± 0.012
Korsmeyer–Peppas	*K_P_* *n* *r^2^*	4.795 ± 1.007 0.501 ± 0.052 0.988 ± 0.007	6.200 ± 1.161 0.491 ± 0.068 0.976 ± 0.020	6.665 ± 1.578 0.509 ± 0.077 0.979 ± 0.015
Peppas–Sahlin	*K_1_* *K_2_* *m* *r^2^*	6.284 ± 0.260 −0.150 ± 0.013 0.450 ± 0.000 0.993 ± 0.003	8.380 ± 0.620 −0.233 ± 0.044 0.450 ± 0.000 0.983 ±0.007	9.384 ± 0.680 −0.232 ± 0.024 0.450 ± 0.000 0.994 ± 0.003

## Data Availability

Not applicable.
